# Supplementary material for: Machine Learning Accelerates Crystallization for Structure Determination
Source: Angew Chem Int Ed Engl. 2026 May 4;65(25):e1218503. doi: 10.1002/anie.1218503 (PMC13266958; doi:10.1002/anie.1218503)

checkCIF/PLATON report

Structure factors have been supplied for datablock(s) lcz090

THIS REPORT IS FOR GUIDANCE ONLY. IF USED AS PART OF A REVIEW PROCEDURE FOR PUBLICATION, IT SHOULD NOT REPLACE THE EXPERTISE OF AN EXPERIENCED CRYSTALLOGRAPHIC REFEREE.

No syntax errors found. CIF dictionary Interpreting this report

Datablock: lcz090

Bond precision:	C-C = 0.0165 A		Wavelength=1.54184
Cell:	a=14.0984(2)	b=21.3196(3)	c=25.4948(3)
	alpha=90	beta=98.138(1)	gamma=90
Temperature:	100 K		

	Calculated	Reported
Volume	7585.86(18)	7585.86(18)
Space group	C c	C 1 c 1
Hall group	C -2yc	C -2yc
Moiety formula	C15 H3 Ag3 F18 N6, C18 H13 N S	C15 H3 Ag3 F18 N6, C18 H13 N S
Sum formula	C33 H16 Ag3 F18 N7 S	C33 H16 Ag3 F18 N7 S
Mr	1208.20	1208.20
Dx,g cm-3	2.116	2.116
Z	8	8
Mu (mm-1)	13.997	13.997
F000	4656.0	4656.0
F000'	4679.64	
h,k,lmax	17,27,32	17,26,32
Nref	16283[8148]	9807
Tmin,Tmax	0.152,0.061	0.358,1.000
Tmin'	0.024	

Correction method= # Reported T Limits: Tmin=0.358 Tmax=1.000
AbsCorr = MULTI-SCAN

```
Data completeness= 1.20/0.60       Theta(max)= 78.388

R(reflections)= 0.0371( 9644)                  wR2(reflections)= 0.1044( 9807)

S = 1.053                Npar= 1118
```

The following ALERTS were generated. Each ALERT has the format
test-name_ALERT_alert-type_alert-level.
Click on the hyperlinks for more details of the test.

Alert level C

```
PLAT090_ALERT_3_C Poor Data / Parameter Ratio (Zmax > 18) ........       6.99 Note
PLAT213_ALERT_2_C Atom F01F             has ADP max/min Ratio .....        3.5 prolat
PLAT213_ALERT_2_C Atom F02D             has ADP max/min Ratio .....        4.0 prolat
PLAT213_ALERT_2_C Atom F032             has ADP max/min Ratio .....        3.5 prolat
PLAT213_ALERT_2_C Atom N00T             has ADP max/min Ratio .....        3.1 prolat
PLAT213_ALERT_2_C Atom C02S             has ADP max/min Ratio .....        3.3 prolat
PLAT220_ALERT_2_C NonSolvent   Resd 2  F   Ueq(max)/Ueq(min) Range         4.8 Ratio
PLAT234_ALERT_4_C Large Hirshfeld Difference F032     --C03A     .        0.24 Ang.
PLAT234_ALERT_4_C Large Hirshfeld Difference C02Q     --C02S     .        0.19 Ang.
PLAT234_ALERT_4_C Large Hirshfeld Difference C03B     --C027     .        0.16 Ang.
PLAT342_ALERT_3_C Low Bond Precision on  C-C Bonds ..............    0.01652 Ang.
PLAT911_ALERT_3_C Missing FCF Refl Between Thmin & STh/L=    0.600         26 Report
                0  2  0,   7 11  0,   1 13  0,   0 22  0,  -1  1  1,   1 13  1,
               -3  1  2,   6 10  2,   5 11  3,  16  0  4,  -7 23  4,  15  9  5,
               -6 22  5,  -6  0  6,  -8 22  6,   8  2  8,  -9 21  8,   0 22 10,
                1 23 11,   4  0 12, -11 17 13,   2 22 13,   7 13 20,   7 13 21,
                6 14 21,  -5 17 22,
```

Alert level G

```
PLAT003_ALERT_2_G Number of Uiso or U(i,j)  Restrained non-H-Atoms         1 Report
PLAT083_ALERT_2_G SHELXL Second Parameter in WGHT  Unusually Large     39.85 Why ?
PLAT186_ALERT_4_G The CIF-Embedded .res File Contains ISOR Records         1 Report
PLAT242_ALERT_2_G Low    'MainMol' Ueq as Compared to Neighbors of       C02U Check
PLAT242_ALERT_2_G Low    'MainMol' Ueq as Compared to Neighbors of       C02X Check
PLAT242_ALERT_2_G Low    'MainMol' Ueq as Compared to Neighbors of       C03F Check
PLAT242_ALERT_2_G Low    'MainMol' Ueq as Compared to Neighbors of       C031 Check
PLAT242_ALERT_2_G Low    'MainMol' Ueq as Compared to Neighbors of       C035 Check
PLAT242_ALERT_2_G Low    'MainMol' Ueq as Compared to Neighbors of       C038 Check
PLAT242_ALERT_2_G Low    'MainMol' Ueq as Compared to Neighbors of       C02I Check
PLAT242_ALERT_2_G Low    'MainMol' Ueq as Compared to Neighbors of       C02M Check
PLAT242_ALERT_2_G Low    'MainMol' Ueq as Compared to Neighbors of       C02Q Check
PLAT242_ALERT_2_G Low    'MainMol' Ueq as Compared to Neighbors of       C02W Check
PLAT242_ALERT_2_G Low    'MainMol' Ueq as Compared to Neighbors of       C03A Check
PLAT242_ALERT_2_G Low    'MainMol' Ueq as Compared to Neighbors of       C037 Check
PLAT720_ALERT_4_G Number of Unusual/Non-Standard Labels ..........        155 Note
              Ag01    Ag04    Ag06    F00A    F00E    F00H    F00J    F00M
              F00Y    F00Z    F01F    F01J    F02D    F02H    F011    F013
              F015    F016    F019    F020    F032    N00D    N00F    N00G
              N00T    N00V    N01X    C01A    C01U    C02I    C02L    C02M
              C02P    C02Q    C02R    C02S    C02W    C02Y    H02Y    C02Z
              H02Z    C03A    C024    H024    C037    Ag02    Ag03    Ag05
```

```
              F00B    F00O    F00P    F00Q    F00R    F00X    F01B    F01C
              F01H    F01K    F01M    F01O    F01Q    F02N    F02O    F010
              F014    N00I    N00K    N00L    N00S    N01E    N009    C01D
              H01D    C01V    C01Z    C02B    H02B    C02C    C02E    C02U
              C02X    C03F    C023    C025    H025    C030    C031    C035
              C038    S007    N00U    C00W    C01G    H01G    C01I    C01L
              C01P    H01P    C01Y    H01Y    C02A    H02A    C02G    H02G
              C02K    H02K    C02V    H02V    C03D    H03D    C03E    H03E
              C012    C021    C026    H026    C029    H029    C034    H034
              C036    H036    S008    N00C    C00N    C01N    C01R    C01S
              C01T    H01T    C01W    H01W    C02F    H02F    C02J    H02J
              C02T    H02T    C03B    H03B    C03C    H03C    C017    C018
              H018    C022    H022    C027    H027    C028    H028    C033
              H033    C039    H039
PLAT860_ALERT_3_G Number of Least-Squares Restraints ............          8 Note
PLAT883_ALERT_1_G Absent Datum for _atom_sites_solution_primary ..     Please Do !
PLAT912_ALERT_4_G Missing # of FCF Reflections Above STh/L=  0.600       299 Note
PLAT915_ALERT_3_G No Flack x Check Done: Low Friedel Pair Coverage        24 %
PLAT933_ALERT_2_G Number of HKL-OMIT Records in Embedded .res File        10 Note
               -6  4 13,  -8 10  4,  -6  4  6,  -8 10  3,  -2 22-15,   3  1  3,
               -4  8 -4,   0 22-10,   4  0 12,  -1 23-11,
PLAT941_ALERT_3_G Average HKL Measurement Multiplicity ..........        2.4 Low
PLAT969_ALERT_5_G The 'Henn et al.' R-Factor-gap value ..........      3.042 Note
              Predicted wR2: Based on SigI**2  3.43 or SHELX Weight  9.91
PLAT978_ALERT_2_G Number C-C Bonds with Positive Residual Density.         0 Info
```

```
  0 ALERT level A = Most likely a serious problem - resolve or explain
  0 ALERT level B = A potentially serious problem, consider carefully
 12 ALERT level C = Check. Ensure it is not caused by an omission or oversight
 24 ALERT level G = General information/check it is not something unexpected

  1 ALERT type 1 CIF construction/syntax error, inconsistent or missing data
 22 ALERT type 2 Indicator that the structure model may be wrong or deficient
  6 ALERT type 3 Indicator that the structure quality may be low
  6 ALERT type 4 Improvement, methodology, query or suggestion
  1 ALERT type 5 Informative message, check
```

Validation response form

Please find below a validation response form (VRF) that can be filled in and pasted into your CIF.

```
# start Validation Reply Form
_vrf_PLAT090_lcz090
;
PROBLEM: Poor Data / Parameter Ratio (Zmax > 18) ........       6.99 Note
RESPONSE: ...
;
_vrf_PLAT213_lcz090
;
PROBLEM: Atom F01F            has ADP max/min Ratio .....        3.5 prolat
RESPONSE: ...
;
_vrf_PLAT220_lcz090
;
PROBLEM: NonSolvent   Resd 2  F   Ueq(max)/Ueq(min) Range        4.8 Ratio
```

```
RESPONSE: ...
;
_vrf_PLAT234_lcz090
;
PROBLEM: Large Hirshfeld Difference F032     --C03A     .       0.24 Ang.
RESPONSE: ...
;
_vrf_PLAT342_lcz090
;
PROBLEM: Low Bond Precision on  C-C Bonds ...............    0.01652 Ang.
RESPONSE: ...
;
_vrf_PLAT911_lcz090
;
PROBLEM: Missing FCF Refl Between Thmin & STh/L=    0.600         26 Report
RESPONSE: ...
;
# end Validation Reply Form
```

It is advisable to attempt to resolve as many as possible of the alerts in all categories. Often the minor alerts point to easily fixed oversights, errors and omissions in your CIF or refinement strategy, so attention to these fine details can be worthwhile. It is up to the individual to critically assess their own results and, if necessary, seek expert advice.

PLATON version of 17/09/2025; check.def file version of 17/09/2025

---

duplicate check

No duplication found

Datablock lcz090 - ellipsoid plot

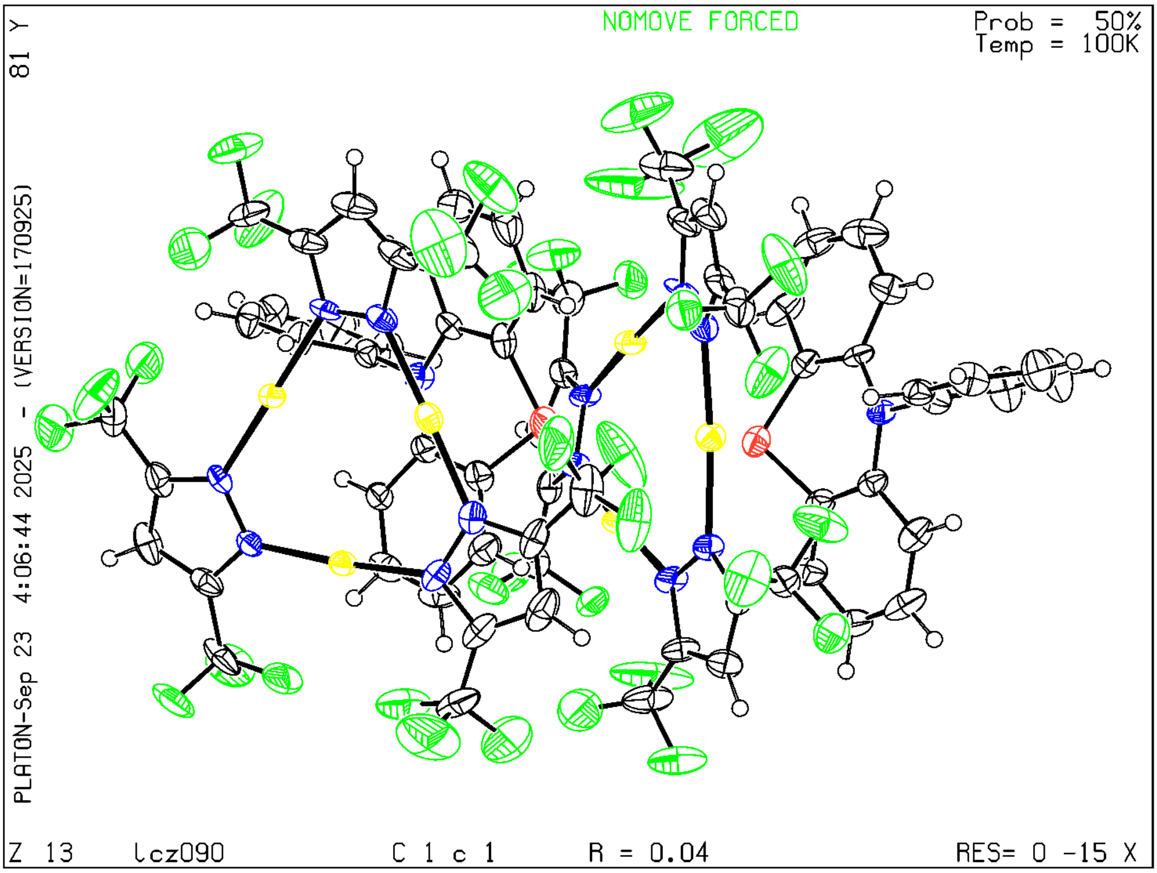

Supplement: Supplementary file 2 — Supporting File 2: anie72462‐sup‐0002‐DataFile.zip. [file ANIE-65-e1218503-s002.zip › supplementary_materials_crystallographic_data submit.zip!/cif_and_checkcif(1-114)/Ag3Pz3_90/Ag3Pz3_90_checkcif.pdf]
